# Study on CerAMfacturing of Novel Alumina Aerospike Nozzles by Lithography-Based Ceramic Vat Photopolymerization (CerAM VPP)

**DOI:** 10.3390/ma15093279

**Published:** 2022-05-03

**Authors:** Eric Schwarzer-Fischer, Johannes Abel, Jan Sieder-Katzmann, Martin Propst, Christian Bach, Uwe Scheithauer, Alexander Michaelis

**Affiliations:** 1 Fraunhofer Institut für Keramische Technologien und Systeme (IKTS), Winterbergstraße 28, 01277 Dresden, Germany; johannes.abel@ikts.fraunhofer.de (J.A.); uwe.scheithauer@ikts.fraunhofer.de (U.S.); alexander.michaelis@ikts.fraunhofer.de (A.M.); 2 Institute of Aerospace Engineering, Chair of Space Systems, Technische Universität Dresden, Helmholtzstr. 10, 01069 Dresden, Germany; jan.sieder-katzmann@tu-dresden.de (J.S.-K.); martin.propst@tu-dresden.de (M.P.); christian.bach1@tu-dresden.de (C.B.)

**Keywords:** alumina, Additive Manufacturing (AM), CerAMfacturing, Vat Photopolymerization (VPP), Digital Light Processing (DLP), Lithography-based Ceramic Manufacturing (LCM), cold-gas nozzle, aerospike nozzle

## Abstract

Advanced ceramics are recognized as key enabling materials possessing combinations of properties not achievable in other material classes. They provide very high thermal, chemical and mechanical resistance and typically exhibit lower densities than metals. These properties predestine ceramics for many different applications, especially those in space. Aerospike nozzles promise an increased performance compared to classic bell nozzles but are also inherently more complex to manufacture due to their shape. Additive manufacturing (AM) drastically simplifies or even enables the fabrication of very complex structures while minimizing the number of individual parts. The applicability of ceramic AM (“CerAMfacturing”) on rocket engines and especially nozzles is consequently investigated in the frame of the “MACARONIS” project, a cooperation of the Institute of Aerospace Engineering at Technische Universität Dresden and the Fraunhofer Institute for Ceramic Technologies and Systems (IKTS) in Dresden. The goal is to develop novel filigree aerospike nozzles with 2.5 N and 10 N thrust. For this purpose, CerAM VPP (ceramic AM via Vat Photopolymerization) using photoreactive and highly particle-filled suspensions was utilized. This contribution gives an overview of the component development starting from CAD modeling, suspension development based on alumina AES-11C, heat treatment and investigation of the microstructure of the sintered components. It could be shown that modifying the suspension composition significantly reduced the formation of cracks during processing, resulting in defect-free filigree aerospike nozzles for application in space.

## 1. Introduction

Technical ceramics are among the most important high-performance materials in all fields of technology. In the aerospace section, the black and white heat protection tiles of the space shuttles are probably the best-known application [1]. Ceramic thermal barrier coatings in metallic rocket engines and turbines have also been established for many years. With additive manufacturing (AM), there is now the possibility of opening completely new forms and fields of application for ceramic materials, as well as new potential for increasing performance and saving mass, which is required for aerospace applications. One starting point, for example, would be the provision of a qualified cold-gas satellite thruster utilizing a ceramic nozzle for future space missions. While conventional engines are mainly made of metals—primarily copper for better heat transfer and nickel as a high-temperature-resistant sheath—advanced ceramics are now also used as a construction material in these areas. Originally, ceramics were only applied as a thermal protection layer, e.g., in the Viking engine of Ariane 4 [2,3,4,5]. In the meantime, however, ceramic matrix composites (CMCs: C/C, C/SiC, SiC/SiC, Al_2_O_3_/Al_2_O_3_, etc.) make it possible to build the entire engine from these lightweight materials. Schmidt et al. [6], for example, were able to successfully test an uncooled rocket engine made of C/SiC for 8900 s. In the field of AM of long-fiber CMCs, Abel et al. [7] investigated SiC/SiC composites manufactured by means of Fused Filament Fabrication.

By AM, various monolithic or graded materials can be produced according to a pixel-by-pixel or line-by-line deposition or curing process. Depending on the principle of the layer building mechanism, not every ceramic powder can be processed. CerAM VPP (pixel-based) and CerAM FFF (line-based) are two representatives of CerAMfacturing and well-known AM processes for ceramic materials. These processes are younger than their polymer counterparts but are already widely known for ceramic materials. They already offer enormous design freedom [8,9,10]. Within the MACARONIS project, a ceramic aerospike engine based on alumina is being designed, manufactured using additive processes and characterized under near-application conditions. 

For this purpose, monolithic ceramic components made of alumina for operation below 1000 °C will first be additively manufactured and tested. Since the various AM processes have different advantages and disadvantages, especially regarding achievable resolution and component size, but also surface quality as well as productivity and costs, the final goal is the hybridization of CerAM VPP and CerAM FFF to combine the advantages of both CerAM processes. Both are used to produce individual parts which are combined by sinter joining, a special joining process during sintering that does not require the use of strength-reducing additives. Sinter joining was developed and successfully patented at Fraunhofer (DE 10 2013 004 807 B4). To enable hybridization, the development of process-specific feedstocks (suspension for CerAM VPP and filaments for CerAM FFF) based on the same alumina powder was necessary. In this paper, only the developments in the field of CerAM VPP are described. The original stereolithography (SLA), invented by Charles W. Hull, is one of the best-known technologies compared to other AM processes and has been already used to produce ceramic green bodies based on alumina (Al_2_O_3_), silicon nitride or silica or for zirconia toughened alumina (ZTA) ceramics [11,12]. The process is characterized by a photosensitive ceramic suspension, which initiates polymerization through partial light exposure, resulting in the solidification of the material. The CerAM VPP technology is derived from stereolithography but has a decisive difference because a digital light processing (DLP) module in combination with a digital micromirror device (DMD) for selective curing is used. One representative of the CerAM VPP technology, which is also used at the Fraunhofer IKTS, is the lithography-based ceramic manufacturing (LCM) technology commercialized by Lithoz (Vienna, Austria) [9]. This technology works with a bottom-up approach and the selective photopolymerization of a highly particle-filled suspension by irradiation via DLP with blue light as a source. 

In this study, the development of a photoreactive suspension for the CerAM VPP process based on alumina, which was also used for the CerAM FFF process [13], is presented. Therefore, three different monomer compositions were compared concerning their flow and curing properties, the printability and the quality of components after thermal processing. The suspension formulation with the best results was used for CerAM VPP of novel alumina aerospike nozzles with a thrust of 2.5 N and 10 N.

## 2. Materials and Methods

The present section describes the utilized materials and methods, beginning with the general design process of the aerospike nozzles in Section 2.1 and a comparison of two nozzle sizes. Subsequently, Section 2.2, Section 2.3, Section 2.4 and Section 2.5 provide an overview of the raw materials, the suspension development including preparation and characterization as well as AM of test components, which were investigated by various methods. In Section 2.6, the “CerAMfacturing” and characterization of aerospike nozzles are described.

### 2.1. Design of Novel Aerospike Nozzles

**Figure 1:**
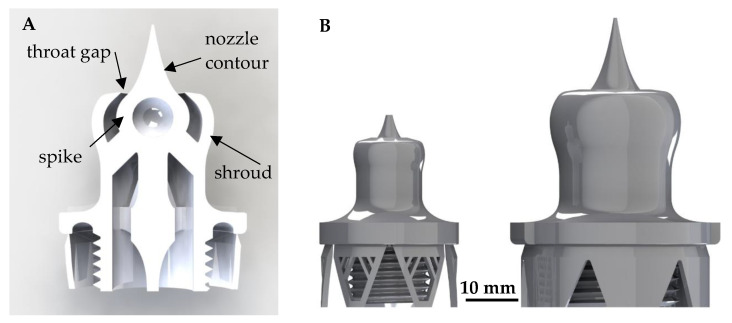
Section display of an aerospike nozzle with necessary needed support structures for AM (**A**) and size comparison of the 2.5 N and 10 N cold-gas aerospike nozzles (**B**).

In order to evaluate the suitability of the above-mentioned AM methods for their applicability in space applications, especially in propulsion systems, aerospike nozzles (see Figure 1) have been chosen, because they promise beneficial performance parameters in comparison to conventional bell nozzles in particular in launch system applications [14,15,16,17,18]. The performance advantage is based on a geometrically free gas expansion over a central body (spike) which allows the adaption of the expansion to ambient pressure. 

In conventional bell nozzles, on the other hand, the supersonic expansion is defined by their fixed outer nozzle contour to a specific nozzle exit pressure, which causes performance losses in an off-design pressure scenario. In other words, the efficiency of bell nozzles depends on altitude.

In this investigation scenario, the aerospike nozzle is used in a cold-gas satellite thruster use-case to focus rather on the design, material and manufacturing challenges than on thermal management. The latter would become dominant in rocket engine applications. Aerospike nozzle designs for the thrust classes 2.5 N and 10 N were derived. An in-house adaption of the nozzle design FORTRAN code of C. C. Lee (BROWN Engineering Company, Des Moines, IA, USA) [19] was used to generate the nozzle contour in the supersonic flow regime. 

The utilized design parameters for the supersonic contour of the nozzles are summarized in Table 1. The parameters were chosen to respect the AM design limitations, such as minimum gap sizes and overhang angles. The sub-sonic flow regime between the threaded connection and the throat gap was designed such that sudden changes in the cross-sectional area and flow tearing edges are avoided and a sub-sonic flow is assured.

As a first orientation, the general key design and manufacturing challenges for the manufacturing can be summarized in the definition of the following requirements:

- Deviations of concentricity between inner spike and outer shroud (<30 µm);
- Dimensional accuracy and roundness of the annular throat gap (<20 µm);
- Low surface roughness (<20 µm);
- Cleanliness (no organic residues, no free particles).

### 2.2. Raw Materials and Suspension Development

As the ceramic raw material, alumina (AES-11C, d_0.5_ = 0.4 µm, Sumitomo Chemical, Tokyo, Japan) with a BET-surface area of 7.66 m^2^/g (measured with HE-pycnometry, ASAP2020, Micromeritics) was used. The used dispersant (Disperbyk 111, BYK-Chemie GmbH, Altana AG, Wesel, Germany) was a solvent-free copolymer with acidic groups, especially a phosphoric polyester with pigment-affine groups usable for the stabilization of inorganic pigments. Various monomers with different functionalities were selected and evaluated regarding their performance. Three different resin compositions (rc) were tested as binder formulation, as can be seen in Table 2.

Camphorquinone was used as the main photoinitiator in combination with ethyl-4 dimethylamino benzoate as a co-initiator (accelerator) to start the radical polymerization of the acrylates after exposure to visible light comparable to the light source of the printing device (wavelength λ = 451 nm). Additionally, polypropylene glycol (PPG, Sigma-Aldrich, St. Louis, MO, USA) was used as plasticizing fluid to reduce the viscosity and the risk of defects due to thermal debinding. Based on the raw materials, photoreactive ceramic suspensions with an alumina powder content of up to 50 vol.% were developed and prepared. 

At first, the photosensitive resin was prepared by mixing the monomers of the resin compositions, the photoinitiator with a content of 1 wt.% and the PPG with a content of 30 wt.% related to the resin by using a planetary centrifugal mixer (Thinky ARV-310, C3-Prozess-und Analysentechnik, Haar, Germany) for 8 min at 2000 1/min. 

To achieve complete dissolution of the initiator, treatment of the premix in an ultrasonic bath was performed, followed by a second mixing step (8 min at 2000 rpm) in the planetary centrifugal mixer. Second, the dispersant and the alumina powder were added to the resin and mixed two times for 10 min at 2000 1/min with the planetary centrifugal mixer. As an intermediate step, the cup with the suspension was dissolved in an ultrasonic bath again for 5 min. 

The optimal dispersant content was investigated for rheological behavior and to achieve low suspension viscosity for the CerAM VPP process to manufacture high-quality alumina components.

### 2.3. Suspension Characterization

The quality of CerAM VPP printed components is fundamentally determined by two essential properties of the used photosensitive suspension. On the one hand, there is the flow behavior, preferably determined by the shear viscosity, and on the other hand, there is the light-induced polymerization of the suspension in dependence on its formulation and light energy used for curing. These two properties were quantified in detail to derive suitable CerAM VPP parameters, such as certain process speeds (e.g., vat rotation) as well as the required energy dose for exposure of one layer (generally 25 µm layer thickness). 

The rheological properties were characterized by measuring the shear viscosity (Modular Compact Rheometer MCR302 with a cone/plate set-up, Anton Paar, Graz, Austria). Viscoelastic behavior (decreasing dynamic viscosity with increasing shear rates) in a low viscosity range (below 100 Pa·s at shear rates up to 1000 s^−1^) is required for use with CerAM VPP due to the rotational suspension coating mechanism of the process. The flow behavior, especially the dynamic viscosity, was measured by stressing the suspension with shear rates in a range of 0.01 to 1000 s^−1^.

The curing by light exposure was determined by measuring the curing depth of a polymerized specimen (approx. 1 mL) depending on the suspension formulation and the used energy dose (smart LED with 450–460 nm wavelength), a value resulting from the irradiation intensity with time (adjusted by a photometer). The layer thickness was measured by using a micrometer (High-Accuracy 13 Digimatic Digital Micrometer, Mitutoyo Europe GmbH, Neuss, Germany). Afterward, the necessary CerAM VPP process parameters were directly derived from the results.

### 2.4. Manufacturing of Test Components via CerAM VPP

Derived from the results of the suspension characterization, a set of processing parameters were used for the manufacturing of the first test samples using the CeraFab 7500 printing device (Lithoz GmbH, Vienna, Austria). Within this process, a layer of the particle-filled slurry is applied by the rotation of a vat in combination with a static wiper blade. The bottom of the vat is transparent so the light source can locally expose the slurry from below. Using a dedicated optical system, the projected image is generated via a DMD with a resolution of 40 µm for the xy-plane, which allows a minimum wall thickness of as low as 100 µm for sintered components. Achievable tolerances are in the range of 40–100 µm, and known roughness *R_a_* values are 0.4 to 2 µm depending on powder type and part orientation. 

**Figure 2:**
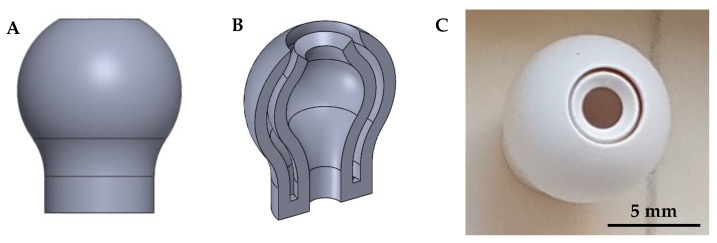
CAD drawing (**A**) with cross-section (**B**) as well as printed (**C**) simplified test specimen.

First, small cubes and tubes were manufactured as test components to evaluate the used printing parameters. Afterward, the three different suspension formulations were used for manufacturing bars (square cross-section of 5 mm edge size and length of 40 mm) and cylinders (diameter d = 2 to 10 mm). In order to quantify the achievable surface properties and manufacturing accuracy, specific test specimens (Figure 2) that represent the geometry of an aerospike engine in a simplified way were manufactured and analyzed.

All manufactured test samples were cleaned with a solvent (Lithasol 30, Lithoz) that dilutes the remaining suspension and solves the monomers, simplifying the removal by pressurized air. 

Following the common ceramic process chain, the cylinders based on the three developed suspension formulations underwent a debinding step (trial and error principle) to remove the polymers of the binder network and all organic compounds by heating them up to 600 °C in a nitrogen atmosphere using slow heating rates (4–10 K/h) with defined temperature holding levels (various dwell times in a range of 2–5 h). 

However, due to nitrogen debinding, some carbon remained in the structure, which was completely removed in a further tempering step up to 600 °C under an air atmosphere. Sintering was performed in an air atmosphere by heating the components up to 1670 °C with a dwell time of 2 h. Heating rates of 1–3 K/min were used with a dwell time of 1 h at 600 °C and 1200 °C. Cooling down to 1200 °C was performed with 0.5 K/min, followed by 1.5 K/min down to 40 °C.

### 2.5. Characterization of Printed Test Components

After cleaning the printed test specimen, an initial green body inspection was carried out regarding the printing quality, i.e., general conditions, edges and surfaces. Therefore, the component quality of the three suspension formulations was compared in the green state and after debinding. 

A detailed investigation of debinding behavior was completed by using thermogravimetric analysis (TGA, heating rate of 1 K/min; up to 900 °C) for suspension formulation rc3 to develop a more efficient heating regime (results presented in Section 3.3). Based on the green and sintered dimensions of the components made of formulation rc3, the sintering shrinkage and the shrinkage correction factors were determined as considered oversize to achieve the correct component dimensions (sintered state), especially the aerospike nozzles. Supporting the sinter shrinkage results more accurately, dilatometric measurements (heating rate of 3 K/min; up to 1670 °C) were conducted in xy- and z-directions.

The density of the sintered samples was calculated according to Archimedes’ principle using purified water, related to the theoretical density (ρ_th_ = 3.92 g/cm^3^) of the raw material. The sintered microstructure was investigated by field emission scanning electron microscopy (FESEM, NVISION 40, Carl Zeiss AG, Oberkochen, Germany).

The surface roughness of the simplified test specimen (Figure 2) was analyzed by measurements with a white light sensor on the MicroProf measuring device from FormFactor (formerly FRT GmbH). A 3 mm measuring head was selected for the measurements, and the samples were each moved under the stationary sensor by means of an automatic xy-table. The step size was 30 µm, and the size of the scan field was 3 mm × 3 mm each. 

### 2.6. Novel Aerospike Nozzles Made by CerAM VPP

Both designs of aerospike trust nozzles were manufactured via CerAM VPP using the Lithoz LCM technology. Therefore, a sinter shrinkage correction factor of 1.22 in the xy-direction and a factor of 1.28 in the z-direction were used with an adjusted exposure energy of 120 mJ/cm^2^ (25 µm layer thickness), a vat rotation speed of 200°/s and a coating thickness of 200 µm. 

Debinding of the nozzles was performed with the developed and improved heating regime followed by sintering similar to the test components. 

After sintering, the quality and dimensions of the nozzles were checked; the dimensional accuracy of the nozzles was partially determined by 3D scans (ATOS core, GOM). For quality assurances, the novel aerospike thrust nozzles were randomly checked concerning possible cracks or defects and other non-visible inner abnormalities by using computed tomography (CT Compact, 180 kV—flat panel detector 5888 × 4600 pixels, Fa. Procon X-ray, Sarstedt, Germany).

## 3. Results and Discussion

This section presents and discusses the results concerning suspension development (Section 3.1) and test component manufacturing (Section 3.2), followed by characterizations (Section 3.3). Afterward, the manufactured alumina aerospike nozzles (Section 3.4) and necessary characterizations (Section 3.4 and Section 3.5) are presented and discussed. 

### 3.1. Suspension Characterization

The Lithoz LCM technology as representative of the CerAM VPP process works with a light-transparent vat, filled with a photosensitive ceramic suspension which is coated by a doctor blade to a thin layer. Standard thicknesses of the coated suspension have values between 100 and 400 µm. During the present work, a value of 225–250 µm was used as coating thickness, which lead to emerging shear rates up to approx. 1000 s^−1^ (200°/s vat rotation) all over the vat in dependence. In general, it is important and advantageous that the viscosity of the used suspension is homogeneous over the upcoming shear range. 

**Figure 3:**
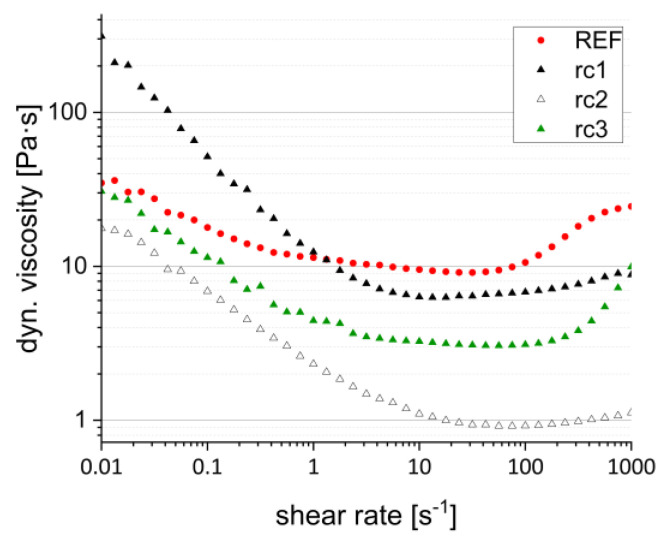
Dynamic viscosity in dependence on shear rate; alumina suspensions with resin composition rc1–rc3.

Figure 3 shows the dynamic viscosity in dependence on shear rate for the suspensions based on the three resin compositions compared to a reference (REF, Lithalox 350) [20]. 

All three developed suspensions generally show a shear thinning behavior, but at different viscosity levels resulting from the different resin compositions. Resin composition rc1 shows the strongest decrease in viscosity changing within two decades by beginning at over 300 Pa·s and reaching 6 Pa·s at low shear rates of 0.01 to 10 s^−1^. Afterward, up to 1000 s^−1^ shear rate, the viscosity follows a nearly Newtonian behavior with 6–10 Pa·s. The behavior at low shear rates is not optimal for the described CerAM VPP process, as the resulting higher viscosity in the middle of the rotating vat as well as during the movement of the building platform (contact and detachment) leads to higher resistance forces, increasing the risk for damage of filigree component areas. Resin composition rc2 shows the same viscosity behavior as resin composition rc1, but at a lower viscosity level ranging between 1 and 20 Pa·s for the entire shear range. The difference from rc1 is that rc2 consists only of low-viscosity monomers, whereas in rc1 a high-viscosity urethane acrylate is used. However, due to the much lower viscosity combined with a narrower range, the influence on the CerAM VPP process is much less compared to the suspension formulation based on rc1. The low viscosity could be also unfavorable, as this reduces the wettability on the surface of the vat. The dewetting favors component defects due to lack of material. Additional additives such as surfactants could help to solve such upcoming phenomena. Resin composition rc3 in that case shows the best rheological behavior with a low viscosity over the whole shear range comparable to the commercial reference. This could be based on the medium viscosity of the monomers, the interaction between the functional groups and the interaction of the functional groups with the surface charge of the alumina particles. Nevertheless, all three suspension formulations were processed in terms of the flow behavior in the CerAM VPP process. 

**Figure 4:**
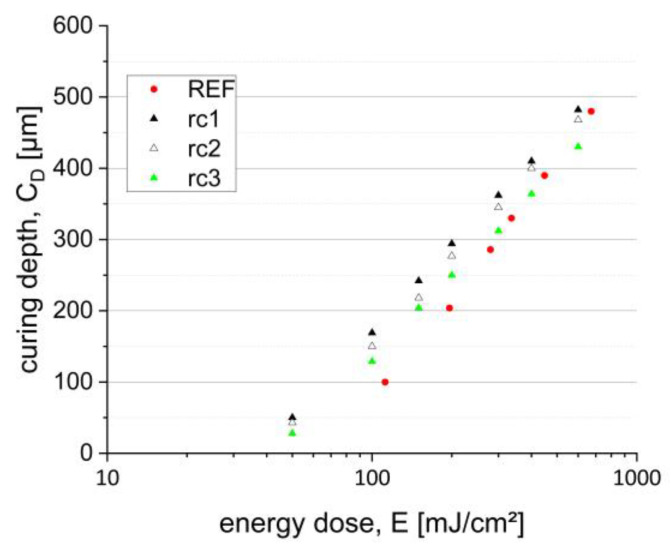
Curing depth in dependence on energy dose; suspensions based on the resin compositions rc1, rc2 and rc3.

A further characteristic important for a successful CerAM VPP processing is the curing behavior, which was analyzed here for all three suspensions. Therefore, the curing depth *C_D_* of a suspension specimen was measured as a function of energy dose. Based on the results, the curing parameters for the CerAM VPP process were derived. Figure 4 shows the curing depth as a function of energy dose for the three suspensions compared to the reference (REF). 

In general, the cure depths of all three suspensions as well as the reference follow the Lambert–Beer law and form as a semi-logarithmic plot a straight line better known as “working curve” [21,22]. There are only minor differences in the curing behaviors. In comparison to the reference, the developed suspensions have in that case a higher reactivity marked by the determined smaller critical energy dose (*E_C_*)—the intersection of the straight lines with the x-axis. This means photolysis starts at a lower energy dose. The difference in the cure depth between the three resin compositions can be explained by the reactivity of the monomers. Due to the chemical structure of rc1, including a large amount of a highly reactive tetrafunctional polyether acrylate, the reactivity is the greatest, followed by rc2, which also includes a tetrafunctional polyether acrylate, but in a smaller amount and combined with a difunctional methylpentanediol diacrylate. As expected, the suspension based on rc3 shows the lowest reactivity visualized by the smallest critical energy dose and slope of the working curve since only a difunctional amine-modified polyester acrylate is used. Achievable cure depths for all three formulations up to a maximum energy dose of 1000 mJ/cm^2^ range between 450 and 480 µm, sufficient for the used CerAM VPP process, which normally works with a layer thickness of 25 µm. Based on the curing depth result, the energy dose for printing should be adjusted in a range of 50 to 130 mJ/cm^2^ depending on the component design. Rough components with large exposure areas should be cured with a lower energy dose, whereas filigree design with small wall thicknesses requires a higher energy dose.

### 3.2. Test Components

**Figure 5:**
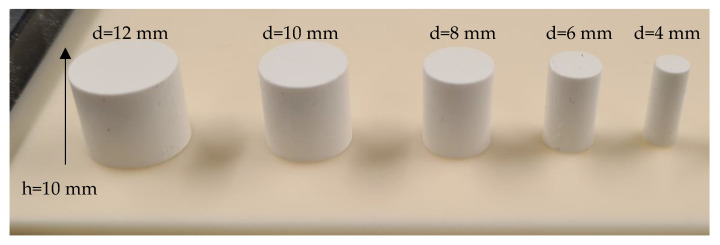
Printed and cleaned cylindrical components; green state with various diameters and a height of h = 10 mm.

After suspension development and characterization, initial printing tests were performed by manufacturing cylindrical components with diameters of 4 mm to 12 mm. The printing parameters were set with a vat rotation of 200°/s and an exposure energy dose of 95 mJ/cm^2^ (rc1), 105 mJ/cm^2^ (rc2) and 115 mJ/cm^2^ (rc3). Figure 5 presents exemplarily printed and cleaned cylindrical green bodies as described. 

Regardless of the resin composition, the three developed alumina suspensions could be printed successfully with the adjusted parameters. After cleaning, the green cylinders were visually inspected for anomalies and possible defects; none were found. Following this initial quality inspection, the diameter and height of the cylinder were measured using a caliper. The actual value and target value matched quite well for all components; measured differences ranged within the tolerance of the caliper accuracy.

**Figure 6:**
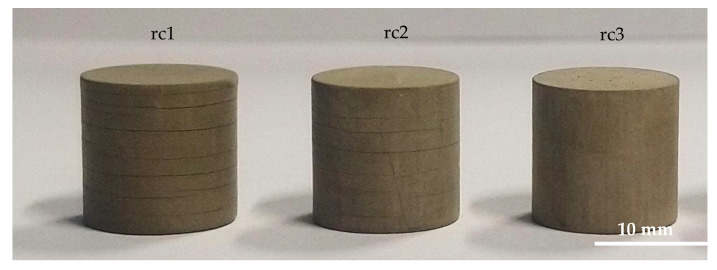
Debinded cylinders (diameter d = 12 mm) of all three developed suspensions.

The next step in the process chain is the debinding, which was first performed in a nitrogen atmosphere as it is known to be much smoother for the components regarding crack formation. A slow heating rate of 10 K/h with a maximum debinding temperature of 600 °C and dwell times of 4 h every 100 °C were used. Figure 6 presents exemplarily a choice of debinded cylinders. 

**Figure 7:**
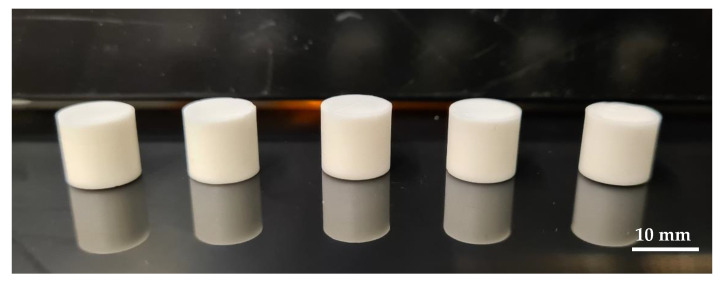
Sintered alumina cylinders (d = 12 mm) based on suspension rc3.

As a result of the debinding, some cylinders showed significant cracking mostly horizontally across the layer interfaces. Eespecially those with a diameter of 12 mm exhibit a high amount of defects. Furthermore, a kind of trend emerged, because the components based on rc1 had the most cracks, followed by rc2 with only a few cracks. Only the components of rc3 had no cracks formed during debinding. Based on this result and the moderate rheological behavior of rc3 suspension compared to the reference, it was decided to focus on this suspension for further steps. However, this does not mean that the other two formulations are unsuitable, but here it is imperative to investigate the debinding behavior in more detail to find the cause of the cracking and to have a chance to avoid crack formation in the future. After debinding, the crack-free cylinders were sintered in an air atmosphere; the result is presented in Figure 7.

As the result shows, the sintering of the cylindric test components with the used heating profile was successful, because no defects could be detected by visual inspection. Further characterizations such as density measurements and microstructure examinations were carried out in the next step and confirmed the first good results. 

### 3.3. Basic Characterization

**Figure 8:**
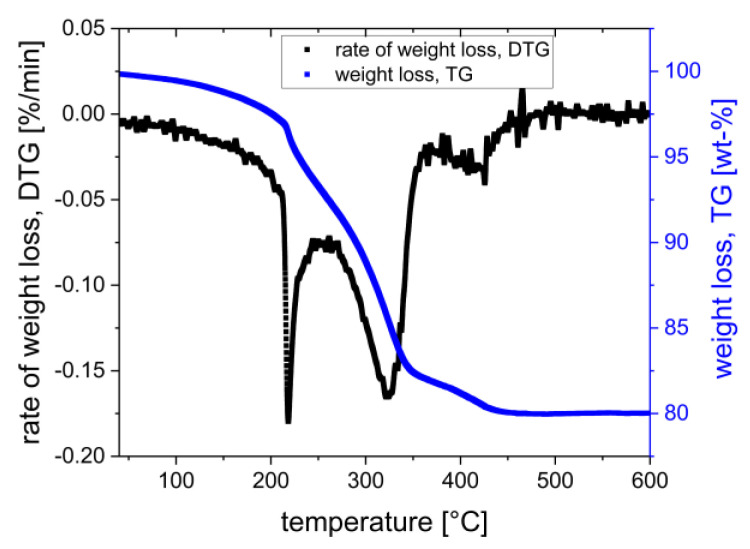
TGA-rate of weight loss and weight loss in dependence on heating temperature.

The first cylindrical test specimens were debinded very slowly for more than 120 h. A future goal is the optimization of debinding, especially the reduction in the debinding time without causing defects such as cracks. Therefore, the debinding of the suspension based on monomer formulation rc3 was characterized in more detail by TGA. The result of the TGA is presented in Figure 8 as a plot of the weight loss ratio and weight loss in dependence on heating temperature. 

**Figure 9:**
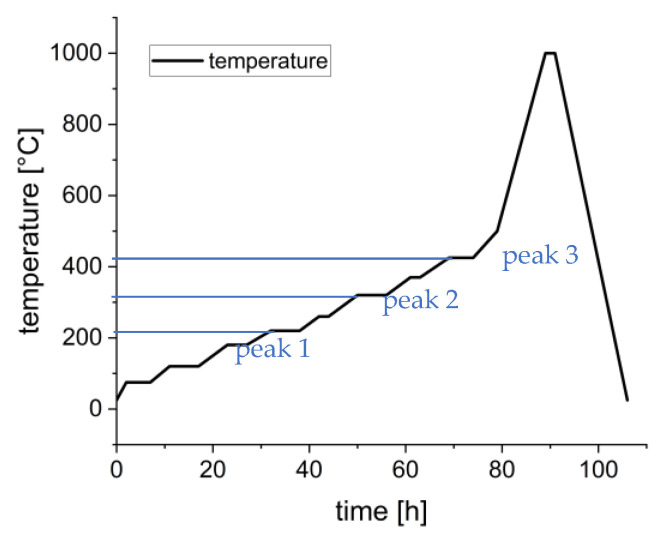
Developed heating profile for debinding based on TGA (Figure 8).

TGA analysis helps to understand the debinding and to find out how much of the binder monomers decompose at which temperatures. Based on the result, it is possible to adjust and optimize the heating profile for the removal of organics because a given TGA plot visualizes when their decomposition occurs and how much of them, especially of a certain component, decompose. Depending on the formulation of the binder, different significant ranges (peak points) of decomposition occur. For the used formulation rc3, a first gentle zone occurs within 100 to 220 °C with a weight loss of up to 7 wt.% (of 21 wt.% complete binder); in that case, the plasticizing fluid (PPG) decomposes. This is one of the critical parts because the PPG starts to evaporate. If the heating rate is too high, in this stage, the binder evaporation leads to crack formation because the diffusion and pore formation is very slow due to the dense green body. The big (negative) increase in the weight loss rate shows the great danger of too much mass decomposition in too little space, in too short a time. It is necessary to heat the sample very gently up to 220 °C. After removing the PPG, a moderate pore space should be created, which is advantageous for removing the other binder components. The next interesting range is within 270–320 °C, also marked by a strong weight loss rate and a weight loss of 7–8 wt.%. Afterward, only a small range of low weight loss occurs at 380–420 °C with an uncritical weight loss of approx. 5 wt.%. Based on the given results, a more specific debinding profile, Figure 9, was developed and used for debinding further manufactured test components based on suspension formulation rc3.

The heating regime for debinding was derived from the TGA. Up to 200 °C, there is a gentle loss of mass; thus, a slightly stepwise heating with rates of up to 10 K/h was applied. Between 200 and 240 °C (peak 1), the mass loss rate increased significantly, so the heating rate was reduced to 4 K/h with increased dwell times of up to 6 h at 220 °C. Up to 300 °C, the heating rate was increased to 10 K/h, with a short dwell time of 2 h at 260 °C and 300 °C. In the range of 300–340 °C (peak 2), the heating rate was decreased again to 4 K/h, with a dwell time of 6 h at 320 °C. In that range, half of the mass decomposed, and at that point, approx. 18 wt.% of the organic content had been removed. Within 415–435 °C, the residual 2–3 wt.% of the polymers decomposed. Here, a decreased heating rate of 4 K/h was used. At 440 °C, the heating rate increased up to 60 K/h until the temperature of 1000 °C was reached, with 2 h dwell time as a pre-sintering step. Cooling was also performed with a rate of 60 K/h. 

**Figure 10:**
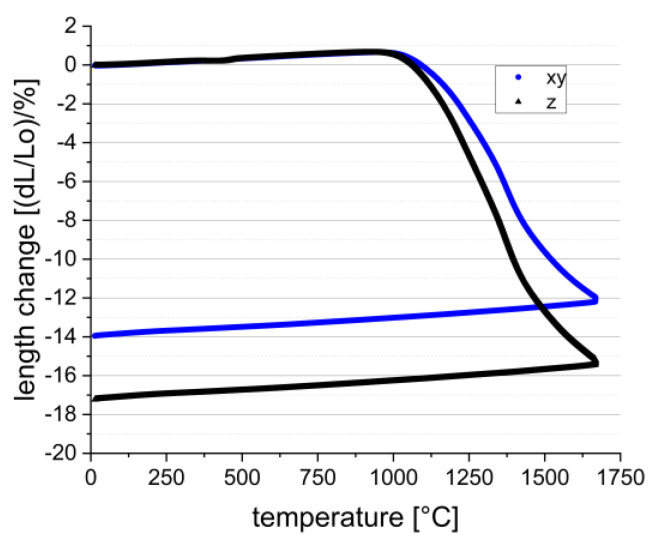
Dilatometric measurements—temperature-dependent shrinkage in xy– and z–directions of the CerAM VPP alumina.

The shrinkage behavior is not less important than the debinding, because on one hand it is necessary to know about the dimensional shrinkage in all dimensions to achieve the correct component sizes after sinter shrinkage, and on the other hand it is important to know at which temperature and time the sinter shrinkage of the material occurs. The second point is very important for the sinter joining approach, an overall objective within the MACARONIS project. The shrinkage behavior must also be known for the sinter joining approach. By dilatometric measurements, the shrinkage in all dimensions was examined. In Figure 10, the result of the dilatometric measurement in the xy- and z-directions is presented. 

As suggested, the results of the shrinkage measurements show a difference of 3% between the xy- and z-shrinkage. This phenomenon is known and seems to be a result of the layerwise building process. In general, the shrinkage values match the expected values for the solid content of 50 vol.%. For dilatometry, debinded components have been used, so a debinding shrinkage of 3–4% had to be added to the measured dilatometric shrinkage. In that case, total shrinkages of up to 18% in xy-direction and up to 21% in z-direction were estimated. To validate the results, the density of the cylinders was measured by hydrostatic weighing with a value of 3.89 g/cm^3^, representing 99.3% relative density compared to the theoretical density of 3.92 g/cm^3^ for the used alumina.

**Figure 11:**
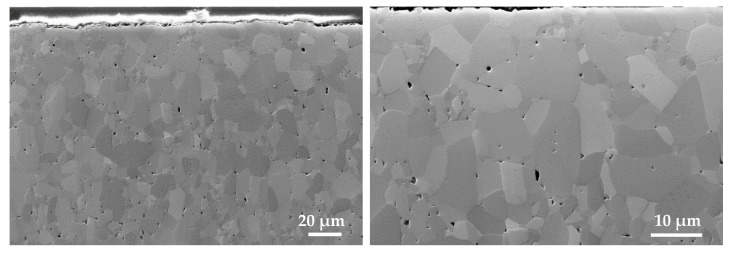
FESEM analysis of the CerAM VPP alumina microstructure, sintered at 1670 °C (2 h dwell).

Finally, the microstructure was analyzed by FESEM, and some images of the result are exemplarily shown in Figure 11.

The result shows a microstructure with good quality, generally comparable to other shaping technologies for the used alumina material. The determined density is consistent with the microstructural images. The number of pores is manageable, but it could be even smaller in the future to achieve an even higher quality. Due to the sintering temperature of 1670 °C and dwell time of 2 h, grain growth can be partially obtained, as can a decrease in grain interfaces marked by blurring and pores within large grains.

**Figure 12:**
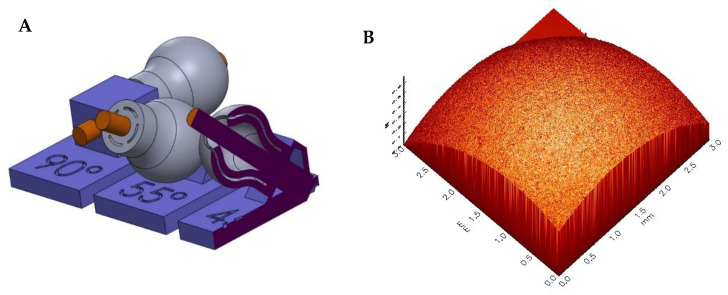
CAD drawing (**A**) of the specimens (grey) fixed on a special holder (blue and orange) to allow the vertical scanning of the surfaces, in that case asintered alumina test specimen; typical image of a surface scan (**B**).

The results of surface roughness as well as the slit width and concentricity analyses are presented in the present section. In order to be able to scan various selected double-curved surfaces, where the cascaded surfaces are most prominent, the specimens were positioned and fixed using a special holder (Figure 12), which in turn was fixed to the moving table. Figure 12 shows a typical image of a surface scan. No cascading surface structures are visible. 

The analysis of the data showed maximum *R_a_* values < 2 µm for all scan areas, in most cases even below 1 µm, where the roughness was determined at five different positions using five differently oriented line scans (0°, 30°, 45°, 60° and 90°).

**Figure 13:**
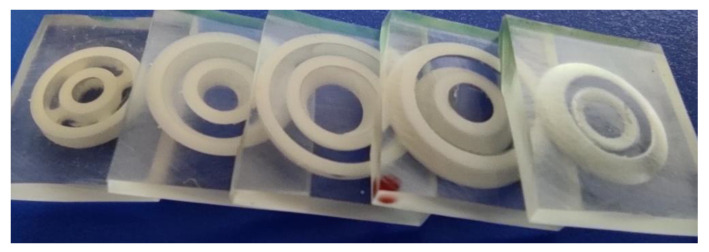
Test specimen embedded in epoxy resin and cut into segments.

Since CT scans are limited in accuracy, a different method was used to analyze slit width and concentricity. Therefore, the surface roughness specimens were embedded in epoxy resin and sawn into individual disc segments (Figure 13), allowing analysis using a light microscope.

Table 3 summarizes the mean values calculated from the measured values and the standard deviation for the five different disc segments. 

The different mean gap widths result from the specimen geometry. The standard deviation is never more than 30.4 µm. Consequently, the sintered specimen reaches a very good concentricity. In conclusion, the required values of concentricity were generally achieved.

### 3.4. Novel Alumina Aerospike Nozzles

**Figure 14:**
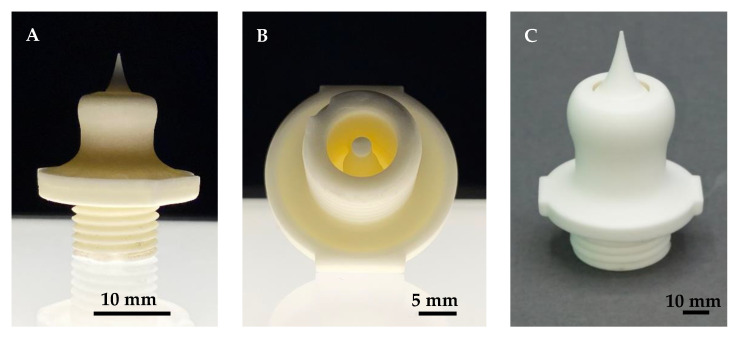
Novel 2.5 N (**A**,**B**) and 10 N (**C**) alumina nozzles additively manufactured using CerAM VPP technology.

After basic developments of the AES 11C-alumina material for CerAM VPP, test printing of the novel aerospike nozzles was performed. Therefore, the printing parameters, especially the layer curing energy, were adjusted to the design. Due to the fact that the sliced nozzle layers consist of small wall thicknesses with low exposure areas, the energy dose of the main layers was adjusted to approx. 100 mJ/cm^2^. The first printing tests show a good result for the chosen parameter set in the green state. After debinding and sintering following the developed heating profile, a good component quality for the 2.5 N and 10 N nozzles was achieved, as can be seen in Figure 14. 

**Figure 15:**
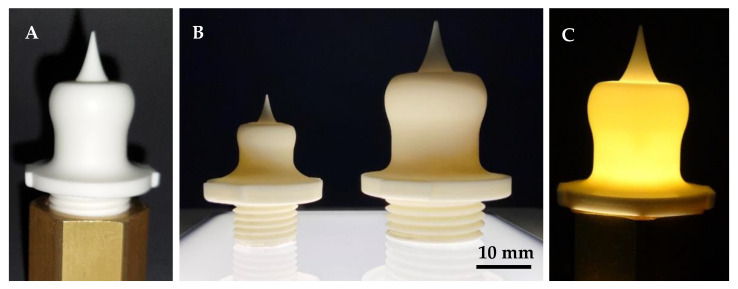
A 10 N nozzle within a metallic counter thread (**A**); visualization of the size difference (**B**); transillumination by lamp for (large) defects (**C**).

After suspension development and parameter optimization, printing and thermal processing of novel alumina aerospike trust nozzles were successful. Components of high quality without visual cracks or any other fatal errors were achieved, and the project goal for single-material AM of demonstrators can be positively concluded. The adjusted shrinkage correction as an oversize factor for printing also matches very well because the fitting accuracy of the thread was given proved by screwing it into a socket with a corresponding metallic counter thread, as can be seen in Figure 15. 

The thread of the nozzles can be fully screwed into the corresponding counter thread (for illustration purposes only it is shown in the half-screwed-in state). In the second image, both nozzle sizes are compared to each other to illustrate the size differences, and the third image shows a transillumination test with a lamp used as the first simple check as screening for large cracks (which have not been detected). 

**Figure 16:**
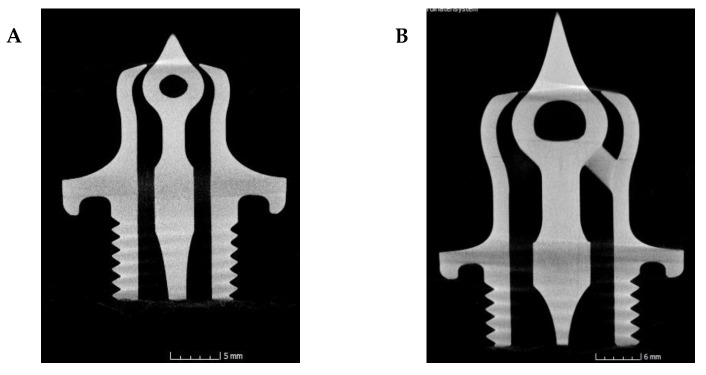
CT images of 2.5 N (**A**) and 10 N (**B**) alumina nozzles.

Since the two nozzle sizes shall be tested regarding their functionality in a special test bench and for safety reasons, a non-destructive characterization for inner cracks and possible inhomogeneity was necessary and was performed using CT analyses; two images of the cross-section are exemplarily shown in Figure 16.

Utilizing the CT images, the result of transillumination by lamp could be confirmed because no cracks or other anomalies were detected. Further, the images show the good quality of the nozzles in general and the surfaces as well as the well cleaned inner part. All channels are open, the narrow gap at the nozzle throat seems to be homogeneous and no essential errors were detected. With this result, the developed nozzles were ready for testing, which is currently being carried out at TU Dresden.

### 3.5. Experimental Cold-Gas and Numerical Flow Characterization

**Figure 17:**
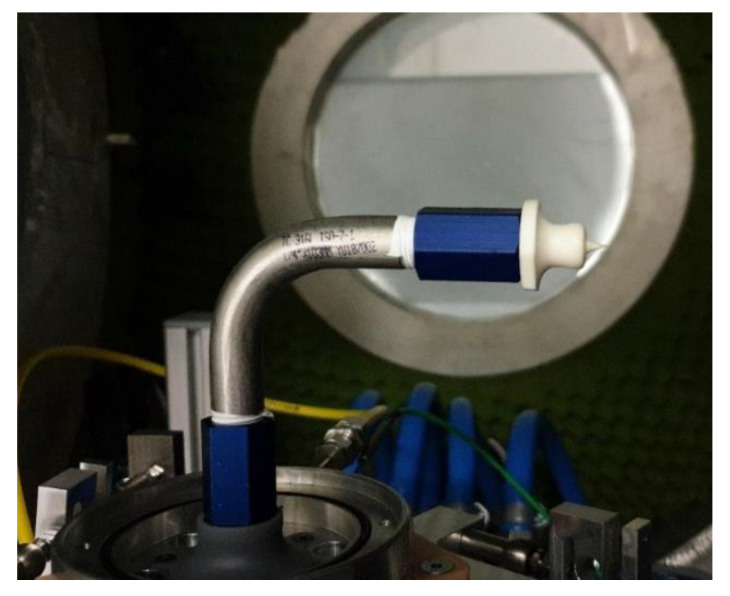
Vacuum test bench for characterization of nozzle flow characteristics; equipped with 2.5 N nozzle.

In order to experimentally determine the flow characteristics and verify the design specifications of the fabricated nozzles, a test bench at TU Dresden is used. An exemplary test assembly is shown in Figure 17. 

This test bench is dedicated to measuring cold-gas nozzles. It is situated in a vacuum chamber, which allows the investigation of higher pressure ratios between the feeding gas (up to 1.1 MPa) and the surrounding atmosphere (down to 5 kPa). A six-degree-of-freedom force balance allows simultaneously measuring all three Cartesian forces and torques; in combination with the measured mass flow, it allows the evaluation of the nozzle performance. The gas properties in terms of temperature and pressure are obtained close to the nozzle in the nozzle holder, which also allows the calculation of the gas density [23,24]. 

Numerical flow analyses utilizing ANSYS Fluent to solve the governing equations are used to investigate the influence of concentricity deviations and the effect of the surface roughness on the nozzle performance and the flow phenomena in otherwise nominal conditions. Deviations of the concentricity could result in a significant asymmetry of the pressure distribution on the nozzle surface, which itself causes undesirable side forces that would have to be compensated. A high surface roughness on the other hand would cause a significant growth of the boundary layer of the flow. That increased boundary layer would act as a partial blocking of the nozzle throat and therefore reduce the mass flow and thrust performance of the nozzle. Hence, the numerical analyses serve as a fundament for evaluating and justifying the quantification of the geometrical manufacturing requirements.

Currently, the additively manufactured 2.5 N and 10 N alumina nozzles are being investigated on the test bench. Preliminarily, for the 2.5 N nozzle type, the design mass flow (3.8 g/s) and the thrust can be compared to the first conducted measurements. Close to the design pressure (201–205 kPa), a mass flow in the range of 3.41–3.59 g/s is measured, resulting in a 5.5–10.2% deviation. A corresponding thrust measurement of 1.93–2.17 N at an ambient pressure of 10 kPa is obtained. A 238 mN difference in thrust is system-immanent due to the non-zero ambient pressure, resulting in a deviation of 3.7–13.3%. The reasons for such significant deviations are still under investigation. Full results and discussion of the experiments as well as numerical analyses will be presented in depth in a dedicated publication focusing on the nozzle flow characteristics and performance.

## 4. Conclusions

Cold-gas aerospike nozzles based on alumina can be one opportunity to maneuver a new generation of satellites in space. CerAMfacturing of novel designs for cold-gas satellite thrusters with 2.5 N and 10 N thrust is presented using CerAM VPP, a ceramic additive manufacturing technology based on stereolithography. For this purpose, the development of a novel photoreactive suspension based on alumina was necessary. Three tailored photoreactive monomer formulations were created and compared across different properties after suspension preparation. The results show a strong influence of the monomers on the flow behavior. In particular, the dynamic viscosity influences the CerAM VPP process directly. A further important effect of the binder formulation could be seen in the debinding results. The formulation is decisive for the success or failure of the debinding step. Strongly cross-linked polymers, which are made of highly functional monomers, have a higher tendency to form cracks. In this case, debinding must be carried out in a much more delicate and gentle manner to avoid the formation of defects. Both aerospike nozzle designs were manufactured by using a suspension formulation based on resin composition rc3. Debinding as well as sintering led to ceramic nozzles of very good quality:

- The measured density reached values above 99% of the theoretical density, which was confirmed using FESEM.
- In addition, the absence of defects in the nozzles was confirmed by means of computed tomography.
- The surface roughness was much lower (*R_a_* < 2 µm) than required, despite the double-curved, layered structures.
- All sintered specimens had a very good concentricity (standard deviation of the gap width < 30 µm).
- Real tests on a vacuum test bench for characterization of nozzle flow characteristics could be realized successfully.

All achieved results form a good basis for the next development steps, such as the hybridization of shaping methods to create larger nozzles. This approach is the combination of components made by CerAM FFF as a filament-based AM method with filigree CerAM VPP components of the same ceramic material via sinter joining without joining additives. This type of hybridization for such nozzles is currently under development.

## Figures and Tables

**Table 1:** Aerospike nozzle design parameters.

Design Parameter	2.5 N Nozzle	10 N Nozzle
throat gap	0.5 mm	1.0 mm
expansion ratio	2.93	2.93
design mass flow (GN2)	3.8 g/s	15.3 g/s
total pressure	203 kPa	203 kPa
nozzle pressure ratio	20.3	20.3

**Table 2:** Components of the tested resin formulations with functionality and content in the mixture.

Variant	Components	Functionality	Viscosity (mPa·s)	Content (wt.%)
rc1	polyether acrylate	4	160	50
	aliphatic urethane diacrylate	2	21 × 10^3^	30
	isobornyl methacrylate	1	12	20
rc2	polyether acrylate	4	100	30
	methylpentanediol diacrylate butandiol monoacrylate	2 1	9 10	20 50
rc3	polyester acrylate (amine modified)	1	150	40
	acrylic acid ester	2	5	60

**Table 3:** Summary of slit width mean values calculated from the measured values.

Segment No.	Average Slit Width (µm)	Standard Deviation—Slit Width (µm)
1	439	14.2
2	474	21.8
3	591	30.4
4	526	26.4
5	612	26.7

## Data Availability

The data are not publicly available due to data confidentiality resulting from the requirements of the accreditation.
